# Current Approaches to the Storage, Processing, and Quality Assessment of RBC and Platelet Components: A Narrative Review

**DOI:** 10.3390/jcm15218222

**Published:** 2026-10-22

**Authors:** Snezhanna Kandrashina, Mikhail Shvedov, Ekaterina Sherstyukova

**Affiliations:** 1 Federal Research and Clinical Center of Intensive Care Medicine and Rehabilitology, V.A. Negovsky Research Institute of General Reanimatology, 107031 Moscow, Russia; shvedovmike@bk.ru (M.S.); kmanchenko@yandex.ru (E.S.); 2 Department of Medical and Biological Physics, Sechenov First Moscow State Medical University, 119991 Moscow, Russia

**Keywords:** RBCs, platelets, hypoxic storage, inert gases, leukoreduction, pathogen reduction, AFM, SICM, Raman spectroscopy, omics

## Abstract

This narrative review summarizes current findings regarding the storage, processing, and quality assessment of red blood cell, platelet components, and whole blood. Particular attention is given to technologies aimed at preserving cellular and functional properties during storage, such as cold storage, cryopreservation, hypoxic and anaerobic storage, modified gas environments, inert gases, leukoreduction, ionizing irradiation, and pathogen-reduction methods. The literature search was conducted in PubMed, Web of Science, Scopus and eLibrary.ru; it included experimental and clinical studies, reviews, guidelines, and regulatory or technical documents published from 1958 to 2026. Evidence demonstrates that various storage and processing strategies offer distinct advantages and limitations, influencing cellular metabolism, membrane and cytoskeleton organization, morphology, mechanical properties, and hemostatic function. While conventional quality-control parameters provide essential information, they may not adequately capture early structural, biomechanical, and molecular changes associated with storage lesions. Consequently, advanced analytical techniques, including atomic force microscopy, scanning ion-conductance microscopy, Raman spectroscopy, metabolomics, and proteomics, are receiving increased attention. These methods facilitate detailed analysis of cell morphology, membrane nanostructure, mechanical properties, hemoglobin status, metabolic changes, and molecular markers of storage-induced damage. Integrating conventional quality-control parameters with biophysical and molecular approaches may enable a more comprehensive evaluation of blood-component quality. Future research should prioritize standardization of emerging storage and analytical technologies, multicenter validation, and identification of parameters associated with post-transfusion recovery and functional efficacy.

## 1. Introduction

Blood transfusion therapy involves the transfusion of whole blood (WB) or its components from a donor to a recipient and remains central to the management of a wide range of clinical conditions [1,2,3]. It is indicated for patients with anemia, coagulopathies, life-threatening bleeding, and chronic diseases, as well as during major surgery, organ transplantation, and anticancer therapy [4,5,6,7,8,9]. Consequently, modern transfusion medicine focuses not only on assuring the availability of blood components but also on improving their safety, standardized quality, and clinically appropriate use [10,11].

Despite its significant clinical value, blood transfusion is associated with various risks [4,12], including allergic reactions [13,14] and infectious complications. To mitigate these risks, ongoing advancements in blood collection, testing, processing, and storage technologies are essential.

Blood services employ molecular methods for infectious disease screening, such as nucleic acid testing to detect pathogen genetic material and minimize the risk of transfusion-transmitted infections [15]. Additional technologies include automated systems for blood collection and component separation [16], leukoreduction and pathogen-reduction techniques [17,18].

Molecular genotyping of donors and recipients represents an additional strategy to enhance transfusion safety. This method complements serological typing, enables the identification of rare and variant antigens, and facilitates the selection of compatible components for patients at elevated risk of alloimmunization [19,20]. High-throughput genotyping further supports the creation of registries of extensively typed donors, improving the matching of red blood cell (RBC) and platelet components [21,22].

Optimizing storage conditions for blood components remains a critical aspect of transfusion medicine [23,24,25]. RBC and platelet components have distinct requirements regarding storage temperature, shelf life, preservative and additive solutions, container materials, and quality-control procedures [5,26]. During storage, cellular blood components undergo metabolic, biochemical, and structural changes that may compromise their functional performance after transfusion [27,28,29]. In RBCs, these changes include depletion of ATP and 2,3-diphosphoglycerate (2,3-DPG), pH alterations, accumulation of metabolic byproducts, hemolysis, vesiculation, and modifications in cell morphology and mechanical properties [30,31]. The extent of these alterations is influenced by storage duration, processing technology, and donor biological variability, such as age, sex, genetic and metabolic characteristics, medication use, and environmental exposures [32,33]. Platelets progressively lose functional activity during storage, and their short shelf life, together with the risk of bacterial contamination, restricts the availability of platelet components [34,35]. Comparative studies have demonstrated differences in microvesicle content between platelet-containing components derived from WB and those obtained via apheresis, including the use of various cell separators [36,37]. Consequently, when evaluating vesiculation, it is essential to consider the method of collection and processing in addition to storage conditions and duration.

In this review, blood-component quality is defined as the set of characteristics that reflect compliance with applicable regulatory requirements and suitability for transfusion. The SQuIPP (Safety, Quality, Identity, Potency, Purity) framework is employed to systematize the assessment of blood components, encompassing safety, quality, identity, biological activity, and purity [38]. Storage and processing technologies are evaluated based on their impact on the safety, purity, and functional preservation of components, while analytical methods are assessed according to the characteristics measured and the extent of clinical validation. Data interpretation requires distinguishing between regulatory quality-control parameters, validated functional metrics, research-grade biomarkers of storage lesion, and experimental analytical measurements. A parameter’s classification depends on the scope of its validation; analytical reproducibility or correlation with storage duration alone does not establish regulatory or clinical significance. Biomarkers indicate specific signs of storage lesions, functional metrics assess cells’ ability to perform their designated functions, and clinical outcomes are measured in the recipient.

Recent research suggests that assessment of RBC and platelet component quality should not be limited to storage duration and standard biochemical parameters. Metabolomic, proteomic, morphological, and biophysical analyses reveal complex structural and functional changes that occur during storage [39,40,41,42]. Although these advanced methods are not yet incorporated into routine quality control, they are increasingly recognized as valuable tools for detailed characterization of storage-induced cellular damage. At the same time, the identification of such changes does not establish their prognostic significance for a specific recipient. A separate evaluation of the relationship with post-transfusion metrics and clinical outcomes is required [30,43].

The relevance of this review arises from the rapid advancement of technologies for the storage, processing, and quality assessment of blood components. In addition to established methods, new storage regimens, pathogen-reduction technologies, and experimental approaches are under active investigation, with considerable variation in their clinical readiness. The application of biophysical, spectroscopic, and omics techniques is also expanding, providing insights into structural and molecular changes that are not captured by traditional quality-control parameters. Despite the high analytical sensitivity of these methods, their findings do not always correlate with post-transfusion recovery or clinical outcomes. Consequently, a comprehensive evaluation of contemporary technologies is warranted, considering both their degree of clinical implementation and the validated clinical significance of the quality parameters employed.

This narrative review aims to summarize and structure current data on storage and processing technologies for cellular blood components and methods for their laboratory quality assessment. The primary focus is placed on RBC and platelet components, while WB is additionally considered in the context of its storage, pathogen reduction, and use in massive transfusion. In this regard, the storage biology of RBC is examined in the greatest detail. Clinical data are considered in relation to specific technologies and the post-transfusion significance of component quality parameters.

## 2. Materials and Methods

This study was designed as a narrative review. The principal criteria of the Scale for the Assessment of Narrative Review Articles (SANRA) were applied in formulating the review objective, conducting the literature search, and appraising sources [44]. The final bibliography comprised 236 publications published between 1958–2026 and 1 methodological article.

The literature search was performed using PubMed, Web of Science, Scopus and eLibrary.ru. Searches were conducted in English and, for eLibrary.ru, in Russian. Search terms included “blood components”, “RBCs”, “erythrocytes”, “platelets”, “blood storage”, “storage lesion”, “cold-stored platelets”, “whole blood”, “cryopreservation”, “hypoxic storage”, “anaerobic storage”, “inert gases”, “xenon”, “leukoreduction”, “irradiation”, “pathogen reduction”, “quality control”, “atomic force microscopy”, “scanning ion conductance microscopy”, “Raman spectroscopy”, “proteomics”, “metabolomics”, “recovery” and “riboflavin”. These terms were used individually and in combination with the Boolean operators AND and OR. The randomized trials comparing fresher with standard-issue RBCs were identified through a supplementary targeted PubMed search using combinations of “age of blood”, “RBC storage duration”, “fresh red blood cells”, and “randomized trial”, supplemented by screening the reference lists of relevant reviews.

The inclusion criteria encompassed original experimental and clinical studies, review articles, clinical guidelines, and regulatory or technical documents addressing the collection, processing, storage, and quality assessment of RBC and platelet components. After duplicate removal, titles and abstracts were screened sequentially, followed by full-text assessment. Publications outside the review scope, conference abstracts, popular science materials, and duplicate publications were excluded (Figure 1).

## 3. Blood Component Storage and Processing Technologies

Current blood-component storage technologies are designed to extend shelf life, minimize storage-induced damage, and maintain the functional activity of cellular components. Beyond standard temperature regimens and additive solutions, ongoing advancements include cryopreservation, modified gas environments, hypoxic storage, and processing technologies that affect component safety and quality during storage. The principal blood components, current storage and processing technologies, and modern approaches to blood-component quality assessment are schematically summarized in Figure 2.

This review defines the level of clinical readiness as follows. Established clinical practice refers to methods incorporated into current guidelines and regulatory procedures, applied routinely or for established indications. Emerging or clinically adopted technologies included methods with regulatory clearance or a documented track record of clinical use in specific jurisdictions and centers, but which have not yet achieved universal adoption or require further evaluation of clinical outcomes. Technologies at the research stage are characterized by an evidence base predominantly limited to in vitro studies, preclinical work, or early clinical observations. The level of clinical readiness is assessed with respect to the specific technology, blood component, and area of application, recognizing that regulatory status and degree of adoption may vary across jurisdictions and should not be extrapolated to other components or protocol modifications. A summary classification is presented in Table 1.

### 3.1. Established Clinical Practice

#### 3.1.1. Leukoreduction as a Strategy to Improve the Quality and Safety of Blood Components During Storage

Leukoreduction is the removal of leukocytes from the donor blood by filtration to reduce the risk of transfusion-related complications [45,46]. It is used to reduce adverse reactions, including febrile non-hemolytic transfusion reactions, alloimmunization against HLA antigens, and the transmission of leukocyte-associated pathogens such as cytomegalovirus. Studies indicate that leukoreduction substantially reduces these risks, particularly in immunocompromised patients and in those needing repeated transfusions [45,47].

Modern leukocyte filtration uses specialized polymer-fiber filters that retain leukocytes through a combination of mechanical trapping and adhesion. These filters reduce residual leukocyte counts to ${<1\times 10}^{6}$ per unit, consistent with international standards for leukoreduced blood products [29]; however, this criterion should not be regarded as a universal threshold value for all jurisdictions and all component types. Filtration efficiency relies on fiber pore size, surface charge, and hydrophilicity, which influence leukocyte adhesion while permitting RBCs and platelets to pass [95,96]. More recent filter designs, including optimization of filtration surface area and the use of biocompatible materials, have improved leukocyte removal while reducing hemolysis and thereby supporting better component quality [97].

Beyond reducing the risk of transfusion complications, prestorage leukoreduction may alter specific aspects of the RBC storage lesion [98,99]. The removal of residual leukocytes and platelets decreases the accumulation of cytokines, proteolytic enzymes, and reactive oxygen species. This reduction is most consistently observed for IL-8 [100,101,102]. Nevertheless, bioactive lipids continue to accumulate following leukoreduction; thus, lower cytokine levels do not indicate complete elimination of the component’s inflammatory potential [103,104].

In vitro studies suggest that prestorage leukoreduction may decrease hemolysis, oxidative and morphological changes in RBCs, and microvesiculation, although its impact on extracellular K^+^ accumulation remains variable [98,105,106,107,108]. However, these primarily experimental results do not demonstrate complete prevention of storage-induced damage or consistent improvement in clinical outcomes [46].

#### 3.1.2. Ionizing Irradiation of Blood Components

Ionizing irradiation of cellular blood components is used to suppress the proliferative capacity of viable donor T lymphocytes, thereby preventing transfusion-associated graft-versus-host disease [50]. Blood components containing viable lymphocytes, particularly RBC and platelet components, are subjected to irradiation. This method is not intended for comprehensive inactivation of infectious agents [51]. Irradiation is not routinely applied to all cellular blood components; rather, it is reserved for specific clinical indications and for recipients at increased risk of transfusion-associated graft-versus-host disease, as outlined in relevant clinical guidelines [52]. Gamma rays or X-rays are utilized as sources of irradiation [52].

The most pronounced effects occur in RBC components: irradiation increases membrane permeability to cations, resulting in rapid accumulation of extracellular potassium. Subsequent storage leads to increased hemolysis and altered distribution of extracellular hemoglobin between free and vesicle-associated fractions [109,110,111,112]. The evidence regarding whether irradiation directly increases microvesicle formation is inconsistent. Some studies report microvesicle accumulation with increasing component age and longer post-irradiation storage, while others find no independent effect of irradiation on vesiculation [110,112]. The magnitude of these changes depends on the age of the RBC component at irradiation and the duration of subsequent storage. Elevated extracellular potassium is particularly important during rapid or massive transfusion in neonates and young children [113,114].

Washing RBC components can reduce the potassium concentration in the supernatant [115]. Additionally, washing RBCs and platelets is indicated to decrease plasma protein content in patients who experience recurrent severe allergic transfusion reactions [116].

The clinical significance of storage lesions is influenced by component age, duration of post-irradiation storage, and both the volume and rate of transfusion [52,113,114]. Irradiating components later in the storage period is associated with increased hemolysis [52,109] and may decrease post-transfusion RBC recovery. In contrast, the effects of irradiation on platelets are less substantial. One experimental study reported that gamma- and X-ray irradiation did not significantly impair the quality parameters of platelets stored at low temperature for up to 21 days [117]. Similarly, a systematic review found that gamma irradiation generally has minimal impact on most platelet quality measures. However, 41 studies assessed only laboratory parameters, and clinical outcomes were evaluated in very few reports. Consequently, the absence of significant in vitro changes should not be interpreted as evidence of full clinical equivalence [118]. In study [117], gamma- and X-ray irradiation were assessed in apheresis platelets stored at low temperature in platelet additive solution E, which limits the generalizability of these findings to other platelet components.

#### 3.1.3. Cryopreservation of RBCs with Glycerol

Cryopreservation employs extremely low temperatures (−80 °C or −196 °C) to maintain the viability of cells and tissues [119]. This technique offers significant potential for RBC components, as it enables timely transfusion while preserving the blood’s oxygen-transport capacity [77,120]. Cryoprotectants play a critical role in this process by minimizing cell damage during freezing and thawing through the inhibition of ice crystal formation and the reduction in osmotic injury [78,121].

A standard approach for RBC cryopreservation utilizes a glycerol-based cryoprotectant, as previously described [53]. The safety of a cryoprotectant is determined by the extent of RBC damage, while its effectiveness is measured by the properties of thawed cells, including membrane integrity, protein activity, rheological behavior, and metabolite concentrations. In a clinical study, Tullis et al. evaluated 94 units of glycerol-cryopreserved and subsequently deglycerolized RBCs and demonstrated that post-transfusion survival, assessed using chromium-51 labeling, was independent of frozen-storage duration of up to 19 months [54].

Another method for addressing storage-induced metabolic alterations is RBC rejuvenation. Incubation with a solution containing pyruvate, inosine, phosphate, and adenine has been shown to increase intracellular ATP and 2,3-DPG levels [122]. This process can be performed prior to cryopreservation.

#### 3.1.4. Clinically Implemented Pathogen-Reduction Systems for Platelets

Bacterial contamination of platelet components stored at room temperature continues to represent a significant infectious risk associated with transfusion. In addition to pathogen reduction, the FDA recommends culture-based bacterial testing, which may be supplemented by repeat or rapid testing depending on the selected control strategy [123].

Pathogen reduction in platelets using INTERCEPT (amotosalen/UVA) and Mirasol (riboflavin/ultraviolet irradiation) has been adopted by several blood services, although authorized processing conditions and the extent of use differ internationally [55,56,57,59,72,124,125,126]. INTERCEPT for platelets has received both FDA approval and CE marking. Switzerland has reported nationwide implementation for all platelet concentrates. Consequently, these systems should be considered clinically implemented technologies within the relevant jurisdictions, rather than solely experimental methods [57,59].

Comparative studies of INTERCEPT, Mirasol, and THERAFLEX UV-Platelets have evaluated pathogen-inactivation capacity and the effects of treatment on platelet quality [72]. However, laboratory parameter comparisons do not demonstrate equivalent clinical implementation or efficacy across all systems.

Rozhkov et al. [126] compared pathogen-inactivation methods using amotosalen/UVA (AS) and riboflavin/UVB (RB) in apheresis platelets stored in 100% plasma. After three days of storage, the RB group exhibited a greater reduction in platelet count and pH, while these parameters remained more stable in the AS group.

Evaluation of platelet pathogen reduction should include separate assessment of laboratory measures of pathogen inactivation, post-transfusion corrected count increment, frequency and severity of bleeding, need for repeat transfusions, and adverse reactions.

### 3.2. Emerging/Clinically Implemented Technologies

#### 3.2.1. Cold Storage of Platelets and Expanded Use of LTOWB

Cold storage of WB is an established technology. Recently, the expanded use of LTOWB has emerged as a clinically implemented approach in contemporary massive hemorrhage management programs [60,61,62,63,64,65,66,127,128,129,130,131,132]. The term “low titer” refers to anti-A and anti-B antibody levels below defined thresholds, which vary among transfusion services. Cold-stored platelets and cold-stored WB are utilized in specific clinical and logistical scenarios; however, they do not serve as universal substitutes for standard blood components across all indications [60,61,62,127,128]. Storage of platelet components at ${1-6\;}^{{}^{\circ}}C$ is considered a viable alternative to conventional storage at ${20-24\;}^{{}^{\circ}}C$, especially for patients with active bleeding. The FDA allows apheresis platelets to be stored at ${1-6\;}^{{}^{\circ}}C$ for up to 14 days when conventional platelets are unavailable, or their use is impractical [60]. Lower temperatures inhibit bacterial growth and alter platelet function. In a randomized crossover study, healthy volunteers received autologous platelets following pharmacological suppression of endogenous platelet function with acetylsalicylic acid. The transfused platelets had been stored either for 7 days at room temperature or for 14 days at ${4\;}^{{}^{\circ}}C$. Platelets stored at room temperature resulted in superior recovery of αIIbβ3-dependent functions, while cold-stored platelets led to a greater increase in thrombin generation [61]. Evaluating the recovery of specific platelet functions in volunteers following pharmacological intervention provides information on functional response, however, it does not directly assess bleeding control in patients.

Clinical evidence supporting the advantages of cold-stored platelets is currently limited. In a multicenter randomized phase II trial, early platelet transfusion in patients with severe trauma was feasible and did not increase the incidence of thrombotic or other adverse events. Mortality at 24 h was 5.9% with cold-stored platelets compared to 10.2% with standard therapy, although this difference was not statistically significant [62]. These findings indicate potential safety and management advantages, but any effect on clinical outcomes requires larger studies.

During cold storage of WB, platelet ability to aggregate declines and several hemostatic parameters are altered within the first 14 days, although clot-forming capacity, as measured by rotational thromboelastometry, remains within the physiological range [127]. The most significant changes occur during this period, including reduced mechanical strength of the clot. Thrombin generation, however, can be maintained during extended storage, regardless of leukoreduction [128].

The relative benefits of WB versus individual blood components continue to be debated in contemporary transfusion medicine, particularly in the context of massive hemorrhage [63]. Historically, WB served as the primary transfusion product [64]. The advent of component therapy facilitated more efficient utilization of donated blood and enabled targeted correction of anemia, coagulopathy, thrombocytopenia, or hypofibrinogenemia [2]. Component therapy allows for the selective administration of RBC-containing components, plasma, platelets, or cryoprecipitate based on the predominant hemostatic disorder, laboratory results, and the patient’s clinical status [129,130,131,132]. Concurrently, there has been renewed interest in LTOWB for massive hemorrhage, as it provides RBCs, plasma, platelets, and coagulation factors [65], streamlines emergency transfusion logistics, and may decrease the total volume of separately administered components [66]. Retrospective data indicate that a higher proportion of WB in massive transfusion protocols is associated with reduced mortality in trauma patients [65]. The selection between WB and component therapy should be guided by clinical context: WB may be advantageous in the initial management of massive hemorrhage, while component therapy remains essential for selective replacement of specific blood components. However, the association observed in retrospective studies does not establish a causal relationship between LTOWB and mortality reduction, as outcomes may be influenced by injury severity, patient selection, and other aspects of the treatment protocol.

#### 3.2.2. Cryopreserved Platelets with DMSO

Cryopreserved platelets with DMSO represent an emerging technology with limited clinical adoption. Their use is restricted to selected programs, and protocols, regulatory status, and clinical implementation differ across countries [67,68,69,70].

Although platelets play a central role in hemostasis and are clinically important for the management of bleeding, their availability is restricted by a short shelf life, typically 5–7 days [67,133,134]. Cryopreservation induces significant alterations in platelet morphology and function [68]. Biochemical analyses of DMSO as a cryoprotectant have demonstrated reduced expression of the GPIbα receptor, which correlates with accelerated platelet clearance from circulation, and increased membrane exposure of phosphatidylserine, contributing to enhanced procoagulant activity. Cryopreserved platelets have been shown to generate thrombin and fibrin more rapidly under both static and flow conditions, potentially increasing their utility in patients experiencing active bleeding, but this finding alone does not demonstrate more effective bleeding control in patients.

Similar to RBCs, the properties of cryopreserved platelets are influenced by the choice of cryoprotectant, with DMSO being the most frequently utilized agent. Standard protocols typically employ 5 to 6% DMSO, however, its use is constrained by dose-dependent effects on platelet function and the potential for adverse reactions following transfusion [69,70].

#### 3.2.3. Platelet Pathogen Reduction with the THERAFLEX UV-Platelets System

THERAFLEX UV-Platelets employs short-wavelength UVC irradiation without the addition of a photosensitizer. CE-marking information has been published for a previous version of the device and its consumables. The technology has also been implemented in a regional German blood service [71]. However, the clinical evidence base and experience with widespread application remain limited.

In the CAPTURE trial, which included 171 patients, non-inferiority of UVC-treated platelets was not demonstrated [71]. This result suggests a potential reduction in post-transfusion platelet increment. However, it does not indicate that the technology is clinically unsuitable.

#### 3.2.4. Pathogen Reduction in Whole Blood with the Mirasol System

The Mirasol system for WB utilizes riboflavin in combination with ultraviolet irradiation to reduce pathogen infectivity and inactivate residual leukocytes. The manufacturer reports that the system holds a CE mark for whole-blood treatment and is available in selected markets [17,72,73,74,75,125]. In the randomized AIMS trial, transfusion-transmitted malaria among initially non-parasitemic patients who received parasitemic blood was observed in 1 of 28 recipients of treated blood compared to 8 of 37 recipients of untreated blood (*p* = 0.039) [76]. These findings indicate a reduction in transmission of this specific infection under the conditions studied.

### 3.3. Research-Stage Technologies

#### 3.3.1. Alternative Cryoprotectants and Experimental Cryopreservation Protocols

Alternative cryoprotectants have not yet demonstrated clinical equivalence to standard glycerol-based cryopreservation. Current research is investigating agents such as dimethylglycine [77], which inhibits ice-crystal growth, maintains water in a bound state, and reduces oxidative cellular damage. Following thawing, RBCs preserved with dimethylglycine display morphological features, osmotic resistance, ATPase activity, Hb content, and erythrocyte sedimentation rates similar to those of untreated cells. Although these results indicate preservation of multiple morphological and biochemical properties after cryopreservation, they do not establish the clinical efficacy or safety of transfusing these cells. Furthermore, a combination of a polyampholyte, dimethyl sulfoxide (DMSO), and trehalose has been proposed to enhance cryoprotection [79]. This formulation reportedly achieved 97% cell recovery after thawing, even under slow freezing and thawing conditions, exceeding the recovery rates observed with glycerol. Other alternative cryoprotectants include small molecules such as trehalose, L-carnitine, and betaine, as well as macromolecules such as hydroxyethyl starch and antifreeze proteins. However, the supporting evidence for these approaches remains largely experimental. Dimethylglycine and the combination of a polyampholyte, DMSO, and trehalose have been evaluated in vitro or ex vivo using isolated sheep RBCs [77,79]. Trehalose-based systems containing membrane-stabilizing glycopeptides or lipids have also been studied in vitro in isolated human RBCs [80,81]. However, no studies involving transfusion to humans have been conducted; thus, preservation of morphofunctional characteristics does not establish clinical efficacy or equivalence to standard glycerol-based cryopreservation.

Saline has been investigated as a non-toxic alternative for platelet cryopreservation [82]. In vitro studies have demonstrated successful cryopreservation of platelets in saline without DMSO, resulting in high cell recovery and maintenance of hemostatic function.

An evaluation of cryopreservation conditions for small platelet volumes found that the type of container influenced the cooling rate but did not significantly affect post-thaw platelet recovery or the preservation of surface receptors GPIbα and GPVI [83].

#### 3.3.2. Modified Gas Mixtures, Xenon, and Other Inert Gases

Storage of blood components in modified gas mixtures and inert gases remains largely experimental in transfusion medicine and is being investigated as a means of prolonging shelf life and preserving blood-cell quality [84,135,136]. Common approaches based on refrigeration and anticoagulants have limitations in retaining the functional activity of platelets and RBCs [137,138,139]. Inert gases may also exert cryoprotective effects by reducing cellular injury at low temperatures [140].

The effect of the gaseous environment on blood-component preservation depends on cell metabolism. Decreasing oxygen availability during RBC storage may limit oxidative damage to hemoglobin, membrane lipids, and proteins of the membrane-cytoskeleton complex [88]. Carbon dioxide can influence the preservation of ATP and 2,3-DPG by altering intracellular pH and the activity of glycolytic enzymes [141,142]. Platelets, by contrast, require enough oxygen supply and effective gas exchange to maintain metabolism, pH, and functional activity [143]. One approach under investigation is the use of inert gases, particularly xenon, to modify the storage atmosphere of blood components.

A protective effect of xenon was not demonstrated during 42-day storage of RBCs [85]. Morphological and biochemical changes were comparable to those observed under standard storage conditions, and hemolysis exceeded the allowable limit, indicating that the gas concentration and storage conditions require further improvement.

Nevertheless, some experimental data show that xenon may have protective effects when applied under increased pressure. During storage of RBCs in a hyperbaric xenon atmosphere [86], hemolysis was comparable to that observed with CPDA-1 preservation and lower than that observed without preservative solution. No major structural or morphological abnormalities were detected, and the proportion of shape-altered RBCs was lower than in the CPDA-1 group.

Hyperbaric storage of platelets with xenon and oxygen at 4 bar has been evaluated as a method for extending storage to 14 days [87]. This approach, however, did not improve platelet function compared with conventional cold storage and introduced additional logistical complexity because it required a pressure chamber and controlled gas removal.

The results of the gas-environment studies cited above primarily characterize the biochemical, morphological, and functional preservation of components in vitro. They do not establish post-transfusion cell recovery or survival.

#### 3.3.3. Experimental Hypoxic and Anaerobic Protocols

Another strategy for modifying the gaseous environment is hypoxic or anaerobic storage of RBC components, in which oxygen levels are deliberately reduced and maintained at low levels using an inert gas or a sealed outer enclosure containing an oxygen scavenger [89]. Under controlled conditions, deoxygenation may help preserve energy and redox metabolism as well as the capacity of RBCs to release bound oxygen [88,135]. However, the effects of hypoxic storage are not uniform and depend on the composition of the gas mixture, container gas permeability, and storage duration [90,91]. Suboptimal conditions may result in morphological alterations, reduced deformability, damage to membrane proteins and lipids, increased ion permeability, and hemolysis. Anaerobic storage may therefore preserve RBC quality only when the relevant storage parameters are carefully controlled. The principal characteristics, potential advantages, and limitations of current blood-component storage strategies are summarized in Table 2. Associations between metabolic changes and post-transfusion recovery have been demonstrated for specific markers and models, but these results do not necessarily apply to all hypoxic storage protocols.

#### 3.3.4. Experimental Combination of Partial Leukoreduction and Whole-Blood Pathogen Reduction

Partial leukoreduction followed by treatment of LTOWB with riboflavin and ultraviolet irradiation constitutes a novel investigational strategy designed to reduce leukocyte counts while maintaining platelet function and the hemostatic integrity of WB [48,49].

An in vitro study assessed cellular composition, hemolysis, coagulation parameters, RBC morphology, mechanical properties, and cytoskeletal organization during seven days of storage and following thawing of isolated RBCs. While several quality indicators, including RBC mechanical properties, were maintained, alterations in specific hemostatic and morphological parameters were detected [48,49].

Enhancements in infectious safety may be associated with alterations in the functional characteristics of blood components. Thomas et al. [17] reported that combined leukoreduction and PR of WB reduced several hemostatic parameters, including platelet aggregability, clotting time, and fibrinogen level.

This combined protocol remains experimental, and its clinical efficacy and safety have not yet been established.

#### 3.3.5. Pathogen Reduction in RBCs

Unlike systems for plasma and platelets, pathogen-reduction technologies for RBCs are not yet used routinely. S-303 remains in clinical development, whereas data on UVC are derived predominantly from in vitro studies.

INTERCEPT for RBCs uses amustaline (S-303) and glutathione. This process differs from INTERCEPT technology for platelets and plasma, which is based on amotosalen and UVA [58,93]. An earlier version of the amustaline/glutathione process was modified following the detection of antibodies specific to pathogen-reduced RBCs in two patients, although neither showed clinical evidence of hemolysis [94].

In the phase III ReCePI trial, 581 patients were randomized, of whom 321 received the investigational RBCs. Specific low-titer antibodies were detected in 5 of 159 recipients of treated RBCs, without evidence of hemolysis [58].

In vitro studies of the S-303 system have demonstrated inactivation of several bacteria and viruses, as well as Plasmodium falciparum [144].

Direct pathogen reduction in an RBC concentrate should also be distinguished from the isolation of RBCs from WB previously treated with Mirasol. Authorization of Mirasol for WB does not, by itself, establish the regulatory status of any resulting RBC-containing component. For a specific product, the separation method, additive solution, storage duration, and corresponding clinical validation are relevant.

## 4. Advanced Methods

Routine laboratory quality control of cellular blood components is based on parameters established by the applicable regulatory framework and the specification of the specific component. In the European Union, Annex V to Commission Directive 2004/33/EC specifies, for the listed RBC components, hemolysis <0.8% of RBC mass at the end of storage; for leukoreduced RBC and platelet components, a leukocyte count <1 × 10^6^ per unit or pool; and for platelets, pH ≥ 6.4, corrected to 22 °C, at the end of storage [145]. The EDQM Guide provides harmonized technical standards and recommendations. Its application depends on incorporation into European and national regulatory systems [29]. In the United States, 21 CFR §640.25(b)(2) requires a platelet pH ≥ 6.2, measured at the storage temperature [146]. The set of regulatory parameters and their acceptance criteria depend on the component type, jurisdiction, and specific product specification. Therefore, compliance with a regulatory criterion indicates fulfillment of the established quality-control requirement.

In routine quality assessment of platelet components, the visual swirling test serves as an indirect indicator of the preservation of discoid platelet shape [147]. Due to potential variability in visual interpretation, swirling results are evaluated alongside pH, platelet count, and functional parameters [148]. This test augments standard quality control by providing a morphological assessment, thereby aligning with contemporary approaches for analyzing cellular structural and functional properties.

Storage duration is a regulated technological characteristic of blood components, whereas the biochemical, structural, and mechanical properties of individual RBC units are also influenced by donor- and processing-related factors [30,31,32,33]. During storage, complex metabolic, structural, and mechanical alterations occur, impacting cell shape, membrane integrity, and cytoskeletal organization. These changes, including potentially reversible metabolic alterations and irreversible membrane loss, are collectively termed RBC and platelet storage lesions [30,35,149,150]. Randomized clinical trials (ABLE, RECESS, INFORM, TRANSFUSE, and ARIPI) did not demonstrate significant benefits of transfusing fresher RBCs compared with standard-issue RBCs in the primary clinical outcomes assessed [151,152,153,154,155]. However, these findings do not exclude storage-induced structural and mechanical alterations, which can be detected by AFM, although their clinical significance and association with post-transfusion outcomes require further validation.

The post-transfusion relevance of laboratory quality parameters is evaluated by comparing them with the recovery and survival of transfused RBCs. Twenty-four-hour recovery quantifies the proportion of transfused RBCs remaining in circulation, while survival characterizes the kinetics of their removal from the bloodstream [156]. Although direct assessment of 24 h RBC recovery using chromium-51 or biotin labeling is impractical for routine testing of individual blood units, ATP content, deformability, and morphology may serve as potential surrogate markers, while the proportion of storage-induced microerythrocytes has been shown to correlate inversely with post-transfusion recovery [157,158]. For platelets, assessment of the post-transfusion platelet count increment should be supplemented by evaluation of clinical bleeding, as these measures reflect distinct aspects of transfusion efficacy [159].

To achieve a more detailed characterization of structural, mechanical, and molecular changes during storage, research studies utilize advanced analytical methods. These include atomic force microscopy (AFM), scanning ion-conductance microscopy (SICM), Raman spectroscopy, and omics approaches such as proteomics and metabolomics [160,161,162]. These techniques reveal morphological, biophysical, and molecular alterations that are not fully captured by standard quality-control parameters. Changes in these parameters should not be interpreted as evidence of improved clinical efficacy or transfusion safety unless a confirmed association with post-transfusion or clinically meaningful outcomes is established.

### 4.1. Atomic Force Microscopy for Assessing the Structural and Mechanical State of Cellular Blood Components

AFM is an informative research method for morphological and biomechanical analysis of cells. It generates high-resolution images of cell morphology and facilitates the assessment of membrane roughness, local surface defects, adhesive properties, and cellular elastic modulus [161,163,164,165,166,167]. AFM has applications across diverse fields, such as cancer research [168,169,170], neurodegenerative diseases [171,172,173], connective-tissue mechanics [174], immunology [175,176,177], and hematology [178,179,180].

In transfusion medicine, morphological, nanoscale structural, and mechanical cell parameters are critical because the therapeutic efficacy of cellular blood components depends on both cell number and the preservation of membrane integrity, cytoskeletal structure, and mechanical properties [41,181,182]. RBC deformability is necessary for passage through the microvasculature [183], while platelet function relies on activation, adhesion, and clot formation capabilities [184,185,186]. AFM enables characterization of the topography, nanostructure, stiffness, and adhesive properties of RBCs and platelets, offering additional indicators of cellular blood-component quality [166,187,188,189].

AFM has been most extensively applied to the study of stored RBCs. Sergunova V. et al. [27] investigated conformational and biomechanical alterations in RBCs stored in CPD/SAGM for 42 days. Between 20 and 24 days of storage, discocytes gradually transformed into echinocytes and spheroechinocytes, accompanied by the emergence of topographical membrane defects and cytoskeletal disorganization. By day 42, increases in cytoskeletal pore area and Young’s modulus indicated enhanced cell stiffness and diminished deformability. Lamzin I. and Khayrullin R. [190] reported similar findings, noting a reduction in RBC diameter along with increases in cell height and Young’s modulus during storage.

AFM has been employed to monitor the progression of RBC vesiculation and to visualize the formation of membrane-associated microparticles [191,192]. These alterations were detected before significant reductions in cell deformability, underscoring the potential of AFM to identify early membrane damage during storage.

AFM has also been applied to monitor changes in the mechanical properties of cryopreserved cells [193,194]. In RBCs, AFM has been used to compare post-thaw morphology following immediate and delayed deglycerolization [195]. This application is consistent with the broader use of AFM as a versatile instrument for quantifying the mechanical properties of living cells, analyzing molecular interactions, and monitoring cellular responses to biochemical or mechanical stimuli [196]. Furthermore, AFM is suitable for investigating the mechanical properties of cells and biomolecules, ligand-receptor interactions, protein folding, and the transport of biologically active substances [197].

AFM is also employed to analyze platelets, as it can simultaneously assess cell topography, local stiffness, and adhesive properties. In studies utilizing force-volume imaging, distinct platelet regions exhibited varying mechanical properties: the central region demonstrated lower stiffness, while the periphery was stiffer, consistent with the distribution of the actin network and microtubules [198].

For platelet components, AFM offers significant value by enabling assessment of morphology, local stiffness, and adhesive properties. These parameters are particularly relevant during platelet storage, as the platelet storage lesion encompasses alterations in cell shape, cytoskeletal organization, reactivity, adhesion, activation, and aggregation. Experimental studies have demonstrated that AFM can map forces across the platelet surface, analyze interactions between RGD-modified probes and the platelet membrane, characterize binding of fibrinogen ligands to the αIIbβ3 receptor, and quantify breaking forces between individual platelets at various activation states [199,200,201,202,203,204]. These methodologies may provide a foundation for more detailed evaluation of platelet functional preservation during storage.

Although AFM provides substantial informational value, it is not routinely implemented as a quality-control method in blood services. Major limitations include prolonged analysis times, intricate sample preparation, inability to conduct in vivo assessments, reliance on scanning parameters, and the requirement for standardized protocols [197,205,206]. Consequently, in the reviewed studies, AFM is primarily employed to analyze structural and mechanical changes in cells. Separate validation is necessary to determine whether the measured parameters can reliably predict post-transfusion recovery and clinical efficacy.

### 4.2. Raman Spectroscopy for Assessing Molecular Changes in Cellular Blood Components During Storage

Raman spectroscopy is a vibrational spectroscopic technique that detects inelastically scattered light from molecules within a sample. This label-free method provides detailed information on the molecular composition of cells and biological fluids, such as proteins, lipids, metabolites, and heme-containing compounds [207,208]. Its significance for blood and blood components is based on its capacity to detect changes in hemoglobin, membrane lipids, protein structures, and metabolic products associated with cell damage or storage [209,210].

Raman spectroscopy has been most extensively applied to the study of RBCs, whose spectral profiles are primarily determined by hemoglobin. This technique enables assessment of hemoglobin state, including the relative proportions of oxyhemoglobin and deoxyhemoglobin, accumulation of methemoglobin, and alterations in the protein–lipid environment of the membrane [210,211].

Atkins et al. [212] demonstrated the feasibility of analyzing supernatant from stored RBC components to detect biochemical changes, including lactate accumulation and inter-donor variability in metabolic profiles. Gautam et al. [213] used Raman spectroscopy to evaluate RBCs stored for up to 42 days, identifying spectral changes related to oxyhemoglobin and methemoglobin status, membrane fluidity, and the progression of hemolysis. These findings support the application of Raman spectroscopy for detecting metabolic and molecular-structural features of the RBC storage lesion.

Another area of research involves non-invasive assessment of RBC components through transfusion containers. Vardaki et al. used spatially offset Raman spectroscopy (SORS) to noninvasively analyze RBC concentrates in sealed plastic bags over 42 days of storage. This approach provided reproducible biochemical information without compromising container sterility and detected storage-related changes in hemoglobin status [162]. In a subsequent study, Vardaki et al. used handheld SORS to non-invasively assess RBC components through standard plastic transfusion bags, reporting mean correlation values of 0.9189 for glucose, 0.8627 for methemoglobin, and 0.7504 for oxyhemoglobin between spectral markers and conventional biochemical measurements [214]. However, these correlations do not establish the method’s ability to predict post-transfusion recovery or clinical outcomes.

Raman spectroscopy has also been applied to platelet analysis. Studies using optical-tweezers micro-Raman spectroscopy have demonstrated that individual living platelets can be analyzed and that activated and non-activated cells can be distinguished by spectral features associated with phospholipids and protein components [215]. Raman tweezers spectroscopy has also been used to study interactions between living platelets and various infusion solutions, demonstrating the method’s potential for assessing platelet responses to external physicochemical conditions [216].

Surface-enhanced Raman spectroscopy (SERS), which increases analytical sensitivity, represents another promising approach for evaluating platelet components. Demishkevich et al. investigated storage-induced spectral changes in platelet-rich plasma stored at ${+4}^{{}^{\circ}}C$ and used multivariate statistics to identify spectral features associated with storage duration [217]. These results suggest that Raman-based approaches are useful for molecular characterization of the platelet storage lesion, although direct studies of standard platelet components remain less numerous than studies of RBC components.

### 4.3. Scanning Ion-Conductance Microscopy for Assessing the Structural State of Blood Cells

SICM is a technique for imaging living cells in liquid, enabling examination of the RBC surface without direct contact between the probe and the membrane [218,219]. This capability is critical for RBCs, as their membranes are easily deformed by contact, and cell shape, surface topography, and membrane fluctuations are closely associated with the state of the membrane-cytoskeleton complex. In the referenced studies, SICM was employed to analyze membrane fluctuations, dynamic morphological changes, and local mechanical properties of RBCs [218,219,220,221]. Importantly, these studies examined RBCs obtained from healthy volunteers and analyzed shortly after collection, rather than RBC units stored under blood-bank conditions.

SICM studies have demonstrated that RBC membrane fluctuations can be quantitatively recorded. The amplitude of these fluctuations indicates cellular mechanical stability, whereas reduced fluctuation activity suggests increased membrane stiffness and impaired membrane dynamics [218]. SICM also detects dynamic changes in RBC shape, facilitating the identification of early morphological alterations [219]. These features may be relevant to the investigation of stored RBC components, in which storage lesions are characterized by progressive loss of discocyte shape, echinocytosis, alterations in surface topography, and decreased deformability; however, their applicability to blood-bank-stored units requires direct experimental validation.

Mechanical mapping represents another significant application of SICM-based techniques. These approaches assess local stiffness of the cell surface at the subcellular level. Tognoni et al. employed SICM to investigate the mechanical response of individual RBCs to localized perturbations, demonstrating the method’s suitability for analyzing the deformation behavior of soft cells [220]. Wang et al. achieved submicrometer mapping of RBC mechanical properties using optically correlated SICM, enabling detection of spatial differences in cell-surface stiffness [221]. Methodologically, these studies contribute to the development of low-load SICM approaches to measure subcellular stiffness while minimizing mechanical damage to samples [222].

SICM has also been applied to the study of living platelets. This technique enables quantitative assessment of platelet area, perimeter, circularity, and spreading without the need for fixation or staining [223]. SICM has revealed dynamic surface changes in activated platelets that are associated with cytoskeletal remodeling [224]. Additionally, it has been used to evaluate platelet shape and mechanical properties under altered intracellular signaling conditions [225]. These findings support the utility of SICM for morphological and biomechanical analysis of platelets, although its application to platelet components during storage remains insufficiently studied. The implementation of SICM-derived parameters for quality control of stored blood components necessitates measurement standardization and validation of their association with post-transfusion outcomes.

### 4.4. Omics Technologies

Omics technologies complement microscopic and spectroscopic methods because they can characterize not only the morphological consequences of storage but also the molecular mechanisms of RBC damage [39,137]. These approaches include metabolomics, proteomics, and lipidomics [226,227]. Together, they provide information on changes in energy metabolism, redox balance, the membrane-cytoskeleton complex, and membrane lipid composition that contribute to the storage lesion and may precede marked hemolysis or gross morphological abnormalities [30,228].

Metabolomics is particularly informative for assessing the energetic state of RBCs during storage [228,229]. Functional preservation of RBCs depends strongly on glycolysis, the pentose phosphate pathway, purine metabolism, and antioxidant systems. During storage, ATP levels decrease, acidosis develops, and lactate accumulates; these changes may impair RBC deformability, alter hemoglobin-oxygen affinity, and reduce membrane resistance to damage [41,226]. Consequently, metabolomic analysis is being explored as an approach for the early detection of molecular indicators of RBC damage, potentially supplementing routine quality-control parameters [228].

Proteomics can characterize changes in membrane and cytoskeletal proteins that determine RBC shape, mechanical stability, and deformability. D’Alessandro et al. performed an integrated time-course metabolomic and proteomic analysis of RBCs stored in SAGM. During the first two weeks of storage, levels of glycolytic metabolites increased. After day 14, metabolic substrates were progressively consumed, metabolism shifted toward the pentose phosphate pathway, markers of oxidative damage accumulated, and the membrane proteome changed. Changes in structural membrane proteins were detectable from day 14, including fragmentation of spectrin, band 3 protein, and protein 4.1. By day 21, pronounced shape alterations were present in a substantial proportion of cells [229].

The evidence supporting individual omics approaches is derived from large multicenter studies. For example, the REDS-III RBC-Omics program used metabolomic analysis to identify the influence of collection conditions and additive solutions on inter-center variability in RBCs [230]. These results demonstrate that omics technologies are currently applied for comparative assessments of how processing and storage conditions affect the molecular characteristics of RBC components. Associations have also been reported between specific genetic and metabolic characteristics and post-transfusion changes in hemoglobin and bilirubin levels [231]. Nevertheless, current evidence does not support the use of omics signatures for routine prediction of clinical outcomes.

A key area of interest is the identification of molecular biomarkers that reflect early storage-induced damage in RBC-containing components. Storage-related changes in the levels of specific metabolites, including hypoxanthine, xanthine, malate, and aspartate, correlate with hemolysis parameters and may be considered biomarkers of RBC damage [228]. Omics studies have revealed inter-donor variability in RBC storage lesions, which are associated with factors including donor age and sex, baseline RBC characteristics, genetic variants, metabolic status, medication use, and blood collection and processing conditions [32,33,232]. Interpretation of omics data must account for the possibility that some detected metabolites may originate from diet, medication use, or donor microbiota activity, in addition to intrinsic RBC metabolism [232]. The observed inter-donor variability supports the application of omics methods to elucidate molecular mechanisms underlying differences in RBC susceptibility to storage-induced damage [233]. Although no recipient group in which profile-guided RBC selection improves clinical outcomes has yet been established, prospective validation could initially focus on recipients undergoing large-volume or repeated RBC transfusions and test whether donor- and unit-specific metabolic or morphological profiles predict 24 h RBC recovery or post-transfusion hemoglobin increment. While these findings have informed the development of personalized transfusion medicine, current evidence is insufficient to support routine selection of RBC components based on predicted storability. Apart from established immunohematological matching and component selection according to clinical indications, this approach remains investigational and requires prospective validation of associations with post-transfusion and clinical outcomes [32,234].

In a broader context, omics technologies can be used to compare additive solutions, temperature regimens, hypoxic storage, PR, and other technological interventions that affect RBC preservation [92,230,235]. A particularly promising direction is the integration of metabolomic, proteomic, biophysical, and clinical laboratory data using machine-learning methods. Such analyses can process multidimensional datasets and identify [39]. However, a storage-related change in the level of a protein or metabolite qualifies it only as a biomarker and does not establish clinical utility. Incorporating such parameters into quality control protocols requires standardized methods and threshold values, reproducibility across donors and laboratories, independent prospective validation, and confirmed associations with post-transfusion or clinical outcomes [32,228]. Until these criteria are fulfilled, metabolomic and proteomic signatures should be considered research-grade biomarkers.

Although AFM, Raman spectroscopy, SICM, and omics technologies offer significant analytical value, their implementation in routine quality control is constrained by limited inter-laboratory reproducibility, complex sample-preparation requirements, operator dependence, absence of standardized protocols, and the necessity for specialized equipment [32,197,205,214,228,236,237]. For most proposed parameters, reproducible associations with post-transfusion cell recovery and survival, hemostatic efficacy, or clinical outcomes have not been established. Consequently, these methods should currently be regarded as research or supplementary tools until multicenter standardization and clinical validation are achieved. Furthermore, widespread adoption in blood centers requires evaluation of costs and organizational feasibility under conditions of high sample throughput.

The principal characteristics, potential advantages, and limitations of current analytical methods for investigating blood components are presented in Table 3.

## 5. Unresolved Issues and Directions for Future Research

Key unresolved issues involve identifying biomarkers that consistently predict post-transfusion recovery and clinical outcomes, as well as evaluating the feasibility of individualizing component storage and allocation based on donor characteristics. Technologies such as AFM, SICM, Raman spectroscopy, and omics approaches require inter-laboratory standardization and validation to confirm that measured parameters correlate with clinically meaningful endpoints.

Currently, clinical data are insufficient to support the routine implementation of hypoxic storage, xenon-based storage, alternative cryoprotectants, and novel RBC pathogen-reduction technologies. Consequently, research priorities should include multicenter prospective studies employing standardized protocols that concurrently evaluate laboratory parameters, post-transfusion cell recovery and survival, hemostatic efficacy, adverse reactions, and patient-centered outcomes. The broad scope of storage, processing, and quality-assessment technologies addressed in this narrative review represents a limitation and precludes an exhaustive evaluation of the clinical efficacy of each approach.

## 6. Conclusions

Established and authorized technologies for blood collection, processing, and storage are designed to enhance the safety of transfusion components and maintain their properties. The quality of the final product depends on the combined effects of collection conditions, container systems, solution composition, processing methods, temperature, storage duration, and individual donor characteristics. Consequently, technologies for the collection and storage of blood components should be developed and evaluated with attention to the specific properties of each component. An integrated approach to improving these technologies may reduce component losses and facilitate more efficient utilization of donor resources.

The clinical readiness of the technologies under consideration varies significantly. Leukoreduction, irradiation for established indications, and authorized pathogen-reduction systems for relevant components are integrated into clinical practice. In contrast, some storage and pathogen-reduction methods are only partially implemented, while alternative cryoprotectants, xenon-based approaches, experimental hypoxic and anaerobic protocols, and RBC pathogen reduction remain largely investigational. Additionally, AFM, SICM, Raman spectroscopy, and omics methods are not yet standard quality-control procedures and require standardization, demonstration of inter-laboratory reproducibility, and multicenter as well as clinical validation.

Future research should focus on standardizing new storage technologies and quality-assessment methods, conducting multicenter comparative studies, and identifying parameters linked to post-transfusion recovery and clinical efficacy. Emphasis should be placed on criteria that can predict the functional preservation of blood components prior to clinical application. A promising avenue involves applying AFM, Raman spectroscopy, and omics methods to characterize small batches of blood-component-based products in development, followed by assessment of these methods’ suitability for quality control. Addressing these challenges will establish a more robust foundation for implementing validated innovative approaches in blood-service practice and may enhance the effectiveness of transfusion therapy.

## Figures and Tables

**Figure 1:**
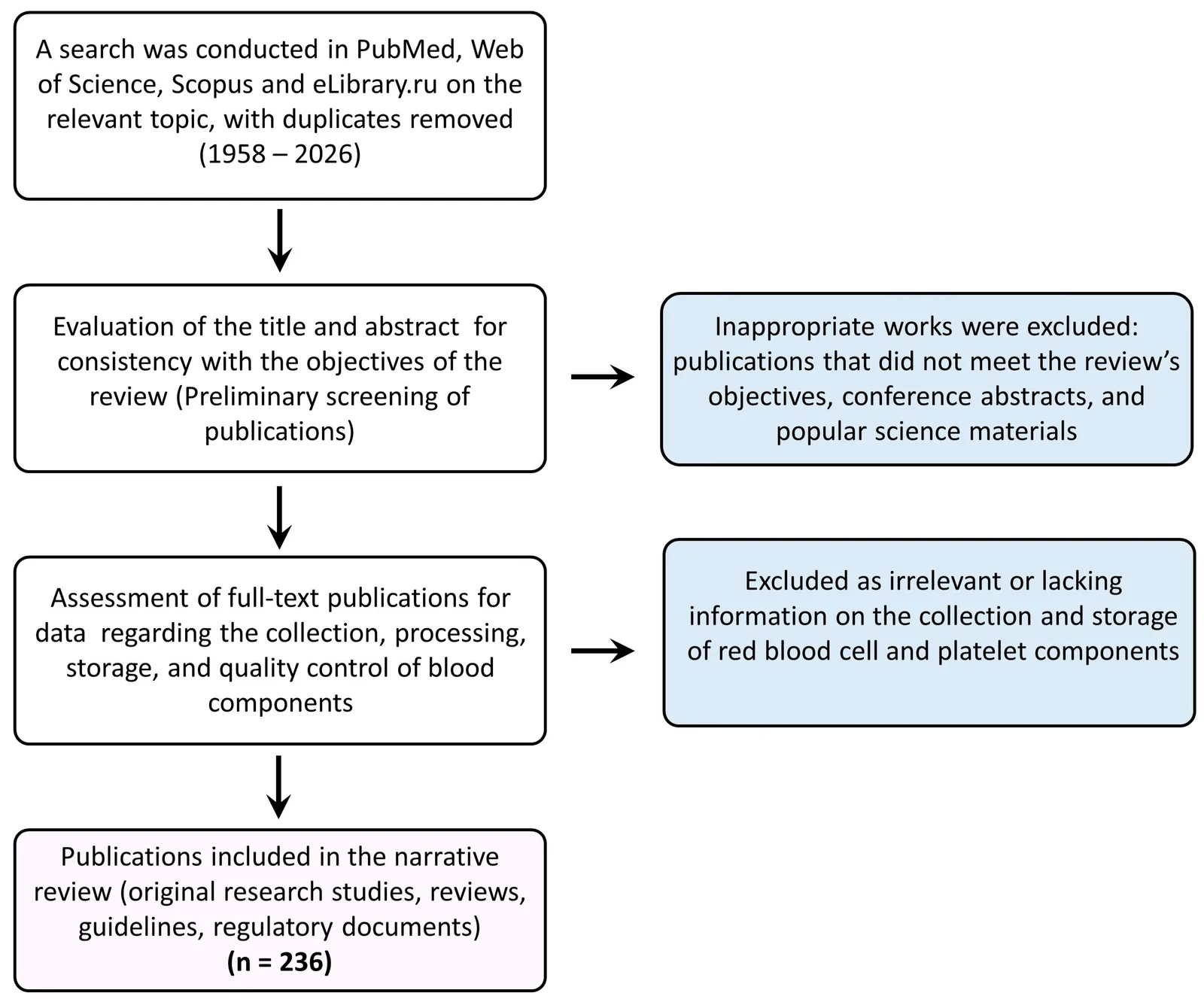
Block-diagram of literature inclusion.

**Figure 2:**
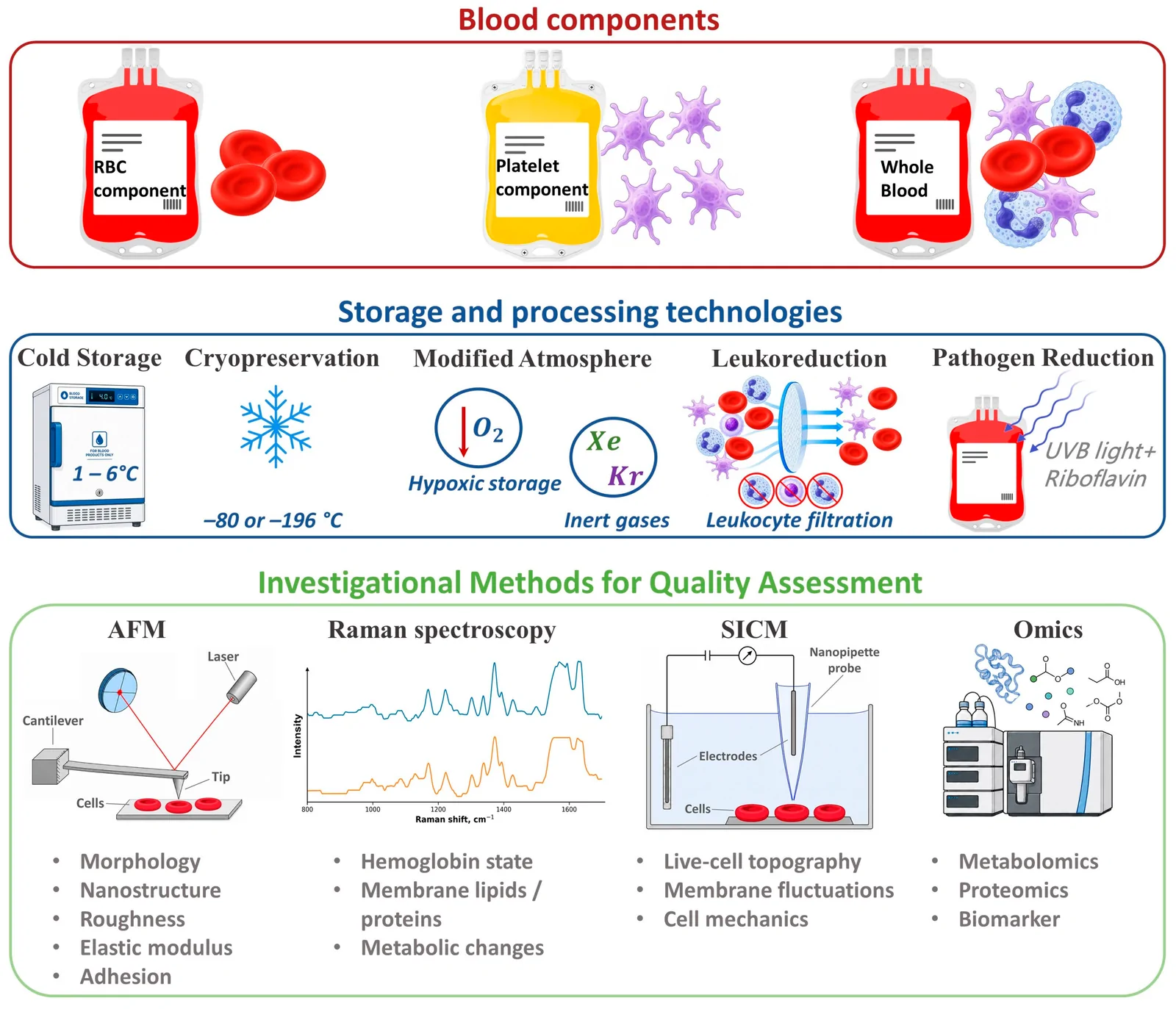
Schematic overview of blood components, current storage and processing technologies, and modern approaches to blood-component quality assessment. The degree of adoption for storage and processing technologies is presented in Table 1. The lower block primarily includes research-grade methods for quality assessment and does not represent an exhaustive list of routine laboratory control methods.

**Table 1:** Classification of Technologies by Level of Clinical Implementation.

	Technology or Protocol	Blood Component	Basis for Classification and Scope of Application	Refs.
Established Clinical Practice	Leukoreduction	Cellular Blood Components	Routine leukocyte removal in accordance with national guidelines experimental partial leukoreduction of Low Titer O Whole Blood (LTOWB) considered separately.	[29,45,46,47,48,49]
	Ionizing Irradiation	RBC and platelets	Clinically applied prevention of transfusion-associated “graft-versus-host” reactions when necessary; not a universal method for inactivating pathogens.	[50,51,52]
	Cryopreservation with Glycerol	RBC	A clinically validated approach to long-term storage, including a rare blood type reserve; deglycerolization is required after defrosting.	[29,53,54]
	INTERCEPT (amotosalen/UVA)	Platelets	Approved and commonly used in several countries; national implementation in Switzerland has been described. This status does not apply to the RBC S-303 version.	[18,55,56,57,58]
	Mirasol (riboflavin/ultraviolet irradiation)	Platelets	Clinical use and published hemovigilance data in countries in which the technology is approved. Approvals and experience with platelets are evaluated separately from those for WB.	[55,56,59]
Emerging/clinically implemented technologies	Cold Storage of Platelets	Apheresis platelets	The FDA permits up to 14 days at 1–6 °C for active bleeding when conventional platelets are unavailable or their use is inconvenient. Not a universal substitute.	[60,61,62]
	Expanded Use of LTOWB	Low Titer O Whole Blood	The inclusion of this approach in treatment protocols for massive blood loss represents a novel advancement in clinical and organizational contexts, rather than in the process of whole blood cooling itself.	[63,64,65,66]
	Cryopreservation with DMSO	Platelets	Limited use in certain programs. Protocols, indications, and regulatory status vary across countries.	[67,68,69,70]
	THERAFLEX UV-Platelets	Platelets	CE marking and details regarding regional implementation. CAPTURE did not demonstrate non-inferior efficacy based on the 1-h CCI. The limitations of the clinical population should be considered.	[56,71]
	Mirasol system for WB	WB	Received CE certification and approval in Ghana, with documented clinical implementation at two centers and within the AIMS study. Implementation remains limited, and there is no marketing authorization in the United States.	[72,73,74,75,76]
Research-stage technologies	Alternative Cryoprotectants	RBC	The substitutes or additives to glycerin under investigation have not had their clinical equivalence to standard cryopreservation established.	[77,78,79,80,81]
	Cryopreservation without DMSO and Experimental Cryopreservation Protocols	Platelets	Laboratory assessment of alternative environments, container types, and sample volumes is necessary, and validation of specific protocols must be conducted.	[82,83]
	Modified Gas Mixtures, Xenon, and Other Inert Gases	RBC and platelets	Experimental conditions for normobaric and hyperbaric storage: inconsistent preservation rates and absence of confirmed clinical benefit.	[84,85,86,87]
	Experimental Hypoxic and Anaerobic Protocols	RBC	The protocols require individual laboratory and clinical validation.	[88,89,90,91,92]
	Partial leukoreduction followed by Mirasol treatment	LTOWB	The modified combination protocol underwent in vitro evaluation. Although Mirasol is approved for use with whole blood, this approval does not establish the clinical readiness of the combined protocol.	[17,48,49]
	INTERCEPT with Amustalin S-303 and glutathione	RBC concentrates	The product is in late-stage clinical development and undergoing regulatory review. Results from the Phase III ReCePI trial are available, and as of 30 July 2026, the product was under review in Europe.	[58,93,94]

**Table 2:** Comparative Characteristics of Current Storage Strategies for RBC, Platelet Components, and Whole-Blood.

Storage Strategy	Blood Component	Principal Conditions	Potential Advantages	Main Limitations and Storage-Induced Changes	Refs.
Cold storage	Platelets	${1-6}^{{}^{\circ}}C$; up to 14 days under specific regulatory conditions	Lower bacterial proliferation; enhanced thrombin generation; potential application in active bleeding	Reduced recovery of some platelet functions; limited clinical-outcome evidence	[60,61,62,139]
	Whole blood	Refrigerated storage; duration dependent on the protocol	Simplification of emergency transfusion; preservation of overall clot-forming capacity	Reduced platelet aggregability and clot strength during storage	[63,64,65,127,128]
Cryopreservation	RBCs	−80 or −196 °C; glycerol or alternative cryoprotectants	Long-term preservation; creation of rare blood reserves	Risk of osmotic injury and hemolysis; labor-intensive deglycerolization; limited clinical validation of alternative cryoprotectants	[29,53,54,78,79,80,81]
	Platelets	Usually 5–6% DMSO; alternative approaches include saline-based protocols	Extended availability; enhanced procoagulant activity	Changes in GPIbα and phosphatidylserine exposure; potential adverse reactions; altered post-transfusion recovery	[67,68,69,70,82,83]
Hypoxic or anaerobic storage	RBCs	Reduced oxygen concentration; oxygen scavengers or sealed outer enclosures	Potential preservation of energy and redox metabolism; reduced oxidative damage	Outcomes depend on gas composition, container permeability, and storage duration	[88,89,90,91,92,141]
Xenon or other inert gases	RBCs and platelets	Normobaric or hyperbaric gas mixtures	Potential reduction in oxidative or low-temperature injury	Contradictory experimental results; additional equipment; no established clinical benefit	[84,85,86,87]

**Table 3:** Comparison of Supplementary Analytical Methods for the Investigation of Blood Components.

Method	Evaluated Parameters	Advantages	Barrier to Routine Implementation	A Potential Role for Quality Control	Refs.
Atomic Force Microscopy (AFM)	Cell morphology; nanostructure and membrane roughness; local defects; stiffness and adhesive properties	High spatial resolution; simultaneous assessment of structure and mechanics; identification of changes in membranes and the cytoskeleton	Time-consuming analysis; sample preparation requirements; dependence of results on measurement parameters; the need for standardization	Supplementary assessment of the structural and mechanical integrity of RBCs and platelets; detection of damage not reflected by routine quality parameters	[27,160,161,166,188,190,191,195,206]
Raman Spectroscopy	Molecular composition of cells and supernatant; hemoglobin status; membrane proteins and lipids; metabolic changes	Analysis with no labels or dyes; molecular specificity; the ability to perform noninvasive analysis of RBC through the storage bag when using SORS	The complexity of interpreting spectra; equipment and standardization requirements; for platelet components, the evidence base is limited	Monitoring of storage-related molecular changes, including hemoglobin status and metabolic shifts; promising approach for non-invasive quality control	[162,209,212,213,214,215,216,217]
Scanning Ion Conductance Microscopy (SICM)	Cell shape and topography; dynamic fluctuations in the membrane surface; local mechanical properties; intracellular reactive oxygen species	Non-contact analysis of live cells in their environment; no direct mechanical interaction with the probe; capability for dynamic measurements	High requirements for equipment and qualifications; limited standardization; the use of this method for storing platelet components has not been sufficiently studied	Supplementary assessment of the morphological and mechanical integrity of living cells and early storage-induced membrane alterations	[218,219,220,221,222,223,224,225,236]
Metabolomics	Energy metabolism; glycolysis; pentose phosphate and purine metabolism; redox status; metabolite profile	Comprehensive assessment of metabolic status; identification of early changes and inter-donor variability; search for potential biomarkers	High cost; difficulties in standardization and data processing; requirement for specialized analytical platforms; need for clinical validation	Identification of metabolic markers of storage-induced damage and potential predictors of the functional preservation of blood components	[32,33,92,228,230,231]
Proteomics	Protein composition; membrane and cytoskeletal proteins; signs of oxidative and structural damage; changes in protein interactions	Systematic characterization of molecular changes; identification of abnormalities in the membrane-cytoskeletal complex	High cost and methodological complexity; labor-intensive data processing; need for standardization and clinical validation	Identification of molecular markers of damage and in-depth assessment of the structural and functional integrity of cells	[137,226,228,229]

## Data Availability

No new data were created or analyzed in this study. Data sharing is not applicable to this article.
